# The Regeneration of Tissue

**Published:** 1899-01

**Authors:** W. P. Whery


### Abstracts and Selections.

### The Regeneration of Tissue.

One of the most interesting and important studies that can engage the attention of the modern surgeon is that of the regeneration of tissues. The great monuments that mark the advances of surgery are placed at the points where the ligature of arteries, the use of anæsthetics, and the aseptic method of operating were introduced in practice. The utilization of the microscope promises to furnish another important landmark in surgery. It will reveal to us the ultimate possibilities in the direction of the regeneration of the tissues. And reinforced with this knowledge surgery will begin a new and grander career of beneficence.

We are familiar with the facility with which the dermal appendages are reproduced. We have experimented a good deal in reproductions of skin, and we get rid of many unsightly scars from burns and ulcers by skin-grafting and other measures promoting a more cosmetic regeneration. The time is, perhaps, coming when the ugly cicatrix will be as obsolete in good surgery as suppuration after operations.

The reproduction or regeneration of the soft parts of the body is not limited to the skin, the muscles, the fascia and ligaments and other fibrous soft tissues. It also includes cartilages and bones, and the methods for effecting their restoration are practiced every day. We cannot very often reproduce a whole bone, unless it be a metacarpal or the terminal phalanx of a finger, but we should not rest content with the present measure of bone regeneration. It has been demonstrated that bony tissue from other animals may be utilized in the replacement of human bone. The surgery of tendons and nerves attracted considerable notice some twenty years ago, but the whole subject is worthy of being studied again with the aid of the microscope.

The frame of the body is not all of it that is amenable to regenerative repair. The journals of medicine contain reports on the repair of brain tissue and the reproduction of genuine cerebral cells to replace the original cells that were destroyed. In the eye an analogous process has been noted. Accounts have also been furnished of the reproduction of the liver tissues, and claim is made that the spleen and kidney can, to some extent, be regenerated after removal by operation of parts of these organs. After oophorectomy, likewise, the ovarian tissue is partially reproduced in many cases.

We need not despair of the regeneration of even the crystalline lens.

The blood is denominated a "fluid tissue;" and it, like the other tissues, illustrates the process of regeneration. We can artificially influence the relative and absolute numbers of the red corpuscles and of the leucocytes, increasing and diminishing them. But it can hardly be said that we have studied the regeneration of the other constitutents to the blood with a view to practical results. There are yet other fluids in the organism that well deserve our most careful research in regard to their regeneration, such as the lymph, the synovia, and the liquor cerebri. Possibly we shall find in these two, material for the reformation of many of the more solid tissues.

In connection with this subject, it is proper to study the physiological equilibrium of the organism. We have several examples of nature's method of substituting one organ, or one process, for another, so that the life and health of the whole body may be continued. For instance, the spleen may compensate for the thyroid gland, or the perspiration process for that of diuresis. Thus it happens that structure may be substituted for structure and function for function, and the observing physician should take advantage of all such facts. The modern views regarding the cell and its contents would largely help the student engaged in research and the practitioner applying the results of such study to the repair of lesions. The essential thing constituting a cell is the specialized protoplasm in the nucleus. The cell wall is of no particular importance. The protoplasm is really and practically the cell, and it is with it that medicine has to deal. Now, the peculiarity of protoplasm is that it possesses a faculty of indefinite vitality under favorable conditions. In such circumstances it might live forever and multiply itself indefinitely. It is this property of protoplasm that makes regeneration of tissues possible, and that renders operative surgery remedial. If with aseptic precautions we could oftener employ the knife to remove diseased or injured tissues, and could manage this in such a way as to allow of the regeneration of the lost parts, we should have achieved the crowning miracle of surgery. To some extent, surgery does accomplish this miracle, and it will have greater triumphs when it employs the microscope before, during, and after its operations as a regular routine. The peculiarities of the process of repair in each class of tissues demand a separate study; or, in other words, the peculiarities of the specialized protoplasm that constitutes the tissue in question. It is often taken for granted that the karyokinesis of all tissue cells is identical. Yet this is very far from being the case. There are as many peculiarities in the physiology of the cells of different tissues in the same body as of the various plants found in the same conservatory. I can here contribute nothing to the science of regeneration, but only offer a suggestion to the student and a prediction of the future of surgery. The details depend on a vast amount of minute work in a variety of fields. —W. P. Whery, in *Medical Times*.

